# Effect of intermittent versus continuous calorie restriction on body weight and cardiometabolic risk markers in subjects with overweight or obesity and mild-to-moderate hypertriglyceridemia: a randomized trial

**DOI:** 10.1186/s12944-020-01399-0

**Published:** 2020-10-07

**Authors:** Mahsa Maroofi, Javad Nasrollahzadeh

**Affiliations:** Department of Clinical Nutrition and Dietetics, Faculty of Nutrition and Food Technology, National Nutrition and Food Technology Research Institute, Shahid Beheshti University of Medical Sciences, Tehran, Iran

**Keywords:** Hypertriglyceridemia, Calorie restriction, Insulin, Obesity, Body weight, intermittent, triglycerides

## Abstract

**Background:**

Intermittent calorie restriction (ICR) is a novel method of dietary restriction for body weight control with the potential to improve obesity-related cardiometabolic markers, but the impact of this diet on subjects with hypertriglyceridemia (HTG) remains unknown.

**Methods:**

Eighty-eight subjects with overweight or obesity and mild-to-moderate HTG were randomized to the continuous calorie restriction (CCR) group, or ICR group (a very low-calorie diet during 3 days of the week) for 8 weeks (44 patients in each group). Body composition, plasma lipids, glucose, insulin, adiponectin, and liver enzymes were measured at baseline and after 8 weeks. An intention-to-treat analysis was performed.

**Results:**

The body weight decreased in both groups (4.07 ± 1.83 kg in the CCR group and 4.57 ± 2.21 kg in the ICR group) with no significant difference between the groups. There was no significant difference between the two groups in the reduced amount of fat mass, fat-free mass, and waist circumference. Both groups achieved a significant reduction in plasma triglycerides after 8 weeks (by 15.6 and 6.3% in ICR and CCR groups, respectively) with no difference between treatment groups. HOMA-IR improved significantly in ICR compared to the CCR group (*P* = 0.03). Plasma glucose, insulin, low-density lipoprotein cholesterol, high-density lipoprotein cholesterol, liver enzyme, and adiponectin were not different between the two groups.

**Conclusions:**

The results of this short-term study suggest that three-days a week of the ICR is comparable to a CCR diet for the reduction of triglycerides level in patients with HTG and in the short-term it appears to be more effective than continuous dieting in improving insulin resistance. However, longer-term studies are needed to confirm these findings.

**Trial registration:**

**Trial registration number:** NCT04143971.

## Background

Obesity is a major concern of global health due to the prevalence of obesity-related disorders, including diabetes and cardiovascular disease [1]. Hypertriglyceridemia (HTG) defined as fasting triglycerides (TGs) ≥150 mg/dL is highly prevalent in the adult population and the prevalence is increasing together with the increasing prevalence of obesity [2, 3]. Mild to moderate HTG is associated with several complications such as cardiovascular disease [3, 4] and pancreatitis [5]. In addition, HTG may play a role in the development of metabolic disturbances of glucose and has been suggested as an independent risk factor for developing impaired fasting glucose or diabetes mellitus [6, 7]. Considering the increased global prevalence of obesity [8], this may indicate a substantial healthcare burden associated with elevated triglycerides (TG) levels [2, 9].

Lifestyle changes including dietary therapy and weight loss could improve HTG [10]. Dietary calorie restriction is a cornerstone of the weight loss program. Continuous calorie restriction (CCR) has been the major form of dietary restriction to reduce weight. However, it may be difficult for some to maintain a constant calorie restriction to achieve weight loss. Intermittent calorie restriction (ICR) is an alternative method of dietary restriction for weight management that involves short periods of severe calorie restriction/fasting interspersed with normal daily caloric intake [11]. The efficacy of ICR for weight reduction is expected since it creates an overall decrease in energy intake.

In HTG, the elevated concentrations of triglyceride levels may lead to peripheral insulin resistance and islet beta-cell dysfunction [12]. Furthermore, obesity is associated with elevated serum free fatty acids which can contribute to insulin resistance in subjects with obesity [13]. The calorie restriction independent of its effect on weight loss could improve insulin sensitivity [14]. Therefore, the calorie restriction method used in the ICR, in which the calorie restriction is more severe on certain days of the week, may have an improved effect on hypertriglyceridemia and insulin resistance.

Previous studies had compared the effects of CCR to ICR on weight loss and cardiometabolic markers in subjects with overweight and obesity [11, 15]. However, currently, there is insufficient data regarding the impact of these diets in subjects with overweight and obesity and mild to moderate HTG. We hypothesized that an ICR diet would result in greater weight loss than a CCR diet in people with overweight and obesity and mild to moderate HTG, and since the calorie restriction in the ICR would be more severe than the CCR diet on some days of the week, the resulting metabolic effects, particularly on plasma TG and insulin concentrations, may also be different.

## Methods

### Study design and subjects

Subjects were recruited through advertisements placed on and around the Nutrition and Diet Therapy Clinic of Shahid-Beheshti University of Medical Sciences and via advertisements in print and broadcast media. Among individuals who responded to the advertisements, 88 participants were deemed eligible to participate after the body mass index (BMI) and plasma TG assessment. Individuals included were men and women with a BMI > 25 kg/m^2^, fasting plasma TG was between 150 and 400 mg/dL in a recent blood test (less than a month ago), who had not had a weight loss program in the previous 3 months and were not taking fibrates or omega-3 supplements, had no previous history of gallstones or cardiac arrhythmia. Exclusion criteria were unwillingness to continue participation, any change in medication regimen during follow up, chronic kidney disease stage 4 or 5, patients with diabetes who were taking insulin or taking insulin-stimulating drugs, currently smoking, and pregnancy. The experimental protocol was approved by the National Nutrition and Food Technology Research Institute and all volunteers gave written informed consent to participate in the trial. The protocol conducted in this trial was in compliance with the Helsinki Declaration and the trial has received ethical approval from the National Nutrition and Food Technology Research Institute Ethics Committee, and written informed consent was obtained from all participants. This clinical trial was registered at the clinical trial registration center (NCT04143971).

The study was a randomized, parallel-armed, comparison between CCR and ICR. Participants were stratified based on BMI (two strata: overweight 25.0–29.9 and obese ≥30.0). Subjects from each stratum were then randomly assigned 1:1 to either the CCR group (*n* = 44) or ICR group (*n =* 44) by the study dietitian according to the randomization schedule. The participants were randomly assigned to either the CCR group (*n* = 44) or ICR group (*n =* 44) following simple randomization procedures using an online-generated random number allocation sequence. The study was not blinded.

### Dietary intervention

Subjects participated in the ICR or CCR protocol for 8 weeks. The CCR group was instructed to consume 70% of the estimated total energy expenditure. For this purpose, first weight maintenance calories were estimated by Mifflin St Jeor’s equation [16] multiplied by physical activity level. To estimate physical activity level, first the short form of the International Physical Activity Questionnaires [17] was completed for each participant and then they transformed to physical activity level (PAL) by using metabolic equivalents to PAL table [18]. The sum of activities value (∆ PAL value) was added to the value of 1.1 (thermic effect of food) and the resulting value was considered as the physical activity level of each participant. Then 30% of the estimated weight maintenance calories were subtracted and the remaining calories were distributed as ~ 52% from carbohydrate, ~ 18% from protein, and ~ 30% from fat. Then, the servings of each food group were determined, and by considering the number of each food group, several meal plans were given to subjects.

Participants of the ICR group consumed a diet that met 100% daily total energy expenditure for weight maintenance (estimated using the Mifflin equation multiplied by physical activity level) during the 4 days of the week, and for the other 3 days, they underwent a fast, consuming a very low-calorie diet (30% of daily calories requirement). A fasting day menu consists of 5–7 servings of meat and substitutes, 0–1 serving of whole grains, 0.5 serving of dairy products, 0–1 serving of fruits, and 5–6 servings of vegetables. Unsweetened tea and coffee were allowed (three servings/day). Participants of both groups also received a general multivitamin/mineral tablet three times during the week.

Dietary counseling sessions were held at weeks 0, 1, 4, and 6 of the study. The dietary counseling sessions were conducted individually for each participant by the study dietitian. For this purpose, the first session of dietary counseling of each participant lasted at least 45 min and the next sessions lasted 15 min. In each session, the participant was learned how to develop individualized meal plans by learning the macronutrient content of food groups and portion sizes. All participants received written dietary information including sample menus and portion advice. No food or meal replacements were provided and all foods were self-selected by participants.

The total weekly calorie of both groups had similar dietary calorie restrictions. A 70% energy restriction 3 days per week with 4 days per week eating at the normal calorie level to maintain weight is equal to a 30% daily energy restriction every day of the week.

The 24-h diet recalls were obtained by a dietitian three times at each of the three-time points (before week 0, at week 4, and week 8 of the study). Dietary data were analyzed by the Nutritionist software (version IV, San Bruno, USA). For the CCR group, the average intake of 3 diet recalls was calculated at each time point. For the ICR group, the diet recalls related to weeks 4 and week 8 were weighted as 3/7 fasting intake and 4/7 fed intake. All dietary information from the diet recalls was analyzed by a single trained operator. Participants were asked not to change their habitual physical activity during the study period.

### Anthropometric measurements

Weight and height were measured during the screening, baseline, and post-intervention visits. The screening visit measurements confirmed BMI eligibility and were not used in the analysis.

Body weight was in light clothing using a balance beam scale (Seca Deutschland, Hamburg, Germany). Waist circumference was measured midway between the lower costal margin and super iliac crest by a flexible tape. Percentage body fat and fat-free mass were assessed after the weigh-in by using a bioelectrical impedance analyzer (X-contact 356; Jawon Medical Co, Seoul, South Korea).

### Biochemical assays

Blood samples were collected into tubes (containing EDTA) after 12 h overnight fasting at the beginning and the end of the study. The platelet-free plasma was separated by centrifugation and stored at − 80 °C for later biochemical analysis. For participants in the ICR group, a final study visit was scheduled to take place the day after the normal-day diet. Plasma glucose, lipids, lipoproteins, and liver enzymes were analyzed with the colorimetry method via auto-analyzer Selectra ProXL (Vital Scientific, Spankeren, The Netherlands), using commercial kits (Pars Azmoon, Karaj, Iran). Commercially available enzyme-linked immunosorbent assay kits were used to measure plasma insulin (Monobind, Inc., Lake Forest, CA, USA) and adiponectin (Biolegend, San Diego, CA, USA) according to manufacturer’s protocol. Insulin resistance was calculated using the homeostasis model assessment of insulin resistance (HOMA-IR) [19].

### Statistics

The study sample size was calculated using weight loss as the main outcome variable. By using mean and the standard deviation (SD) of body weight changes from a previous study [20], to detect an effect size of 0.59 with 80% power at the 5% level of significance, in each arm, 44 participants were needed.

All analyses were conducted using intention-to-treat. The baseline carry forward method was used to manage missing data. Data were analyzed using SPSS (version 23.0, SPSS Inc., Chicago, USA). Kolmogorov-Smirnov test was performed to check the normality of data and to permit parametric testing, non-normally distributed data were log-transformed. Differences between groups were assessed using independent *t*-tests or the *χ* ^2^ test. Between-groups differences were assessed with an ANCOVA test with post-treatment values as the dependent variable, and baseline values of each parameter as the covariate. To present more details, absolute change values from baseline were also analyzed with independent t-tests or non-parametric Manne Whitney U-test. However, since ANCOVA has the highest statistical power [21], the basis for significant differences between the two groups was considered based on the results of the analysis of covariance. Differences between baseline and post-intervention in each group were analyzed by the paired *t*-test. Dietary intake values were assessed with repeated-measures analysis of variance for the two factors with the group as a between-subjects factor. Correlations between changes in outcome variables were explored using Pearson’s or Spearman’s tests. Statistical significance was set at *P* < 0·05.

(two-tailed).

## Results

### Baseline characteristics

**Fig. 1:**
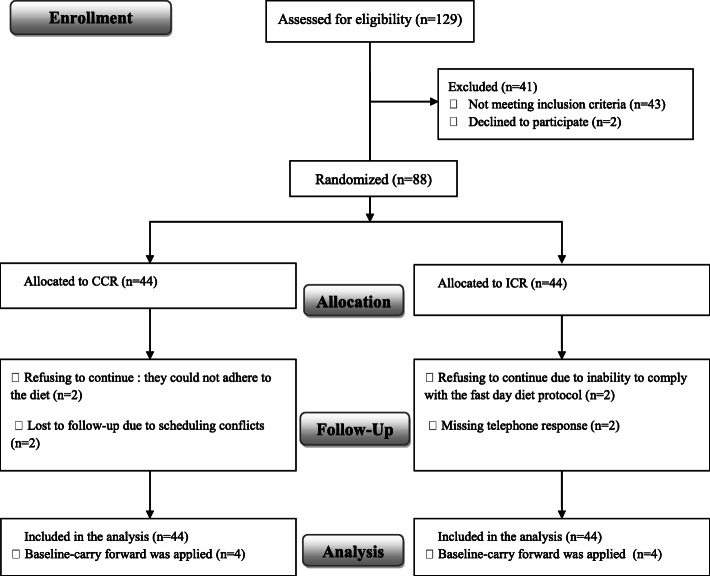
Flowchart of the study

Of the 129 participants screened, 88 adults with HTG were randomly assigned to diet groups and 80 completed the study (Fig. 1). Baseline characteristics of all participants in the CCR and ICR groups are reported in Table 1. There were no differences between groups for age, weight, or medications. Eight participants in the CCR group and 11 in the ICR group were treated with statins. None of the participants were treated with oral estrogen and thiazide drugs before or during the study.

**Table 1:** Baseline characteristics of the participants

Variable	CCR (*n* = 44)	ICR (*n* = 44)	***P***-value
Age (years)	45.2 ± 11.7	44.0 ± 8.6	0.60
Sex (number of men)	15	10	0.34
Weight (kg)	90.1 ± 19.3	83.9 ± 13.7	0.09
BMI (kg/m^2^)	32.4 ± 4.6	31.6 ± 3.9	0.38
Overweight (n)	17	17	1.0
Obese (n)	27	27	
Fat mass (%)	35.9 ± 5.8	37.5 ± 4.6	0.14
Fat-free mass (%)	63.6 ± 5.4	63.0 ± 5.9	0.60
Waist circumference (cm)	104.7 ± 11.0	100.6 ± 9.8	0.067
History of hypertension (n)	5	6	0.52
History of diabetes mellitus (n)	7	5	0.51
Medications			
Statins (n)	8	11	0.60
ACE-I (n)	3	2	0.63
ARB (n)	2	4	0.76
Plasma creatinine (mg/dL)	1.0 ± 0.1	1.0 ± 0.2	0.60
TG (mg/dL)	165.0 (126)	180.5 (115)	0.84
Cholesterol (mg/dL)	190.1 ± 38.1	178.6 ± 30.3	0.12
LDL-C (mg/dL)	107.7 ± 29.0	98.4 ± 28.5	0.13
HDL-C (mg/dL)	42.1 ± 10.6	39.1 ± 7.7	0.13
FBS (mg/dL)	89.5 (16)	88.0 (26)	0.18
Insulin (μIU/mL)	22.0 ± 9.7	18.2 ± 8.1	0.046
HOMA-IR	3.7 (3)	3.5 (3)	0.37

Data presented as mean ± SD, median (interquartile range) or percentages for dichotomous measures

*ACE-I* Angiotensin-converting enzyme inhibitor, *ARB* Angiotensin receptor blocker, *BMI* body mass index, *CCR* Continous calorie restriction diet, *HDL-C* high-density lipoprotein cholesterol, *HOMA-IR* homeostasis model assessment of insulin resistance, *ICR* Intermittent calorie restriction diet, *LDL-C* low-density lipoprotein cholesterol, *TG* triglycerides

### Dietary intake

The dietary intake of participants is shown in Table 2. Participants of both groups ate slightly less than their prescribed goal. There was no between group difference in energy intake. Compared to the CCR group, carbohydrates intakes were significantly lower whereas protein and cholesterol intakes were significantly higher in the ICR group. Data from the dietary recall indicated that participants in the ICR group ate the types and quantities of foods that have been recommended in the fasting days.

**Table 2:** Dietary characteristics of participants during the study ^a^

Variable	Groups						***P***-value ^**b**^	
	CCR (***n*** = 44)			ICR (***n*** = 44)				
	Baseline	Week-4	Week-8	Baseline	Week-4	Week-8	Time*group	Group
**Energy (kcal/day)**	2485.95 ± 418.25	1709.31 ± 362.79	1741.10 ± 346.66	2416.36 ± 393.54	1610.6 ± 239.8	1613.5 ± 245.8	0.72	0.10
**Carbohydrate (% of energy)**	49.71 ± 6.78	52.79 ± 6.04	52.45 ± 7.36	51.98 ± 5.39	47.9 ± 5.1	49.7 ± 5.6	0.001	0.001
**Protein (% of energy)**	15.29 ± 3.22	18.02 ± 2.87	18.50 ± 5.94	15.59 ± 3.22	21.3 ± 2.3	20.8 ± 2.9	0.06	0.001
**Fat (% of energy)**	34.90 ± 5.82	29.10 ± 6.55	29.05 ± 4.68	32.30 ± 6.23	30.9 ± 5.7	29.4 ± 5.3	0.001	0.17
**SFA (g/day)**	22.36 ± 8.68	14.58 ± 3.54	13.39 ± 3.24	19.70 ± 8.24	15.0 ± 3.6	13.8 ± 3.3	0.28	0.11
**MUFA (g/day)**	32.15 ± 12.82	20.09 ± 7.29	20.24 ± 5.26	30.10 ± 11.71	22.6 ± 6.3	21.5 ± 6.1	0.15	0.64
**PUFA (g/day)**	22.49 ± 11.78	14.72 ± 8.75	14.58 ± 8.81	20.15 ± 9.71	15.0 ± 4.7	13.6 ± 4.7	0.42	0.47
**Omega3 (g/day)**	0.724 ± 0.55	0.855 ± 0.56	0.82 ± 0.58	0.932 ± 0.680	0.9 ± 0.4	1.28 ± 2.7	0.50	0.12
**Fiber (g/day)**	20.60 ± 9.59	19.50 ± 8.29	18.41 ± 8.12	19.3 ± 6.6	19.4 ± 6.6	17.8 ± 6.2	0.84	0.57
**Cholesterol (g/day)**	220.52 ± 147.33	204.38 ± 141.48	162.22 ± 110.21	193.63 ± 136.01	274.8 ± 118.0	273.6 ± 151.8	0.002	0.007

^a^All values are mean ± SD

^b^Data were analyzed by repeated-measure ANOVA with the group as a between-subjects factor

*CCR* Continous calorie restriction diet, *ICR* Intermittent calorie restriction diet, *SFA* saturated fatty acid, *MUFA* monounsaturated fatty acid, *PUFA* polyunsaturated fatty acid

### Weight loss and body composition

Both groups lost significant weight between baseline and 8-week follow up (Table 3). Body weight decreased by 4.07 ± 1.83 kg in the CCR group and 4.57 ± 2.21 kg in the ICR group. The analysis showed that the mean weight reduction was not significant by treatment. Waist circumference, Fat mass, and fat-free mass decreased significantly within each group, but there were no significant differences between the groups.

**Table 3:** Body weight and body composition changes during the weight-loss period ^**a**^

Outcome	Groups		***P***-value ^**b,c**^
	CCR (***n =*** 44)	ICR (***n =*** 44)	
**Weight (kg)**			
baseline	90.1 ± 19.3	83.9 ± 13.7	
8-week	86.0 ± 19.2 ^d†^	79.3 ± 13.0 ^†^	0.11
Change	4.1 ± 1.8 ^e^	4.6 ± 2.2	0.25
**BMI (kg/m**^**2**^**)**			
Baseline	32.4 ± 4.6	31.6 ± 4.0	
8-week	31.0 ± 4.6 ^†^	29.9 ± 3.8 ^†^	0.09
Change	1.9 ± 0.6	1.7 ± 0.8	0.13
**Waist circumference (cm)**			
Baseline	104.7 ± 11.0	100.6 ± 9.8	
8-week	100.0 ± 10.7 ^†^	95.0 ± 9.6 ^†^	0.05
Change	4.7 ± 2.4	5.6 ± 3.0	0.12
**Hip circumference (cm)**			
Baseline	112.0 ± 9.8	111.3 ± 9.1	
8-week	109.0 ± 9.9 ^†^	107.8 ± 8.6 ^†^	0.40
Change	3.1 ± 2.8	3.5 ± 2.5	0.45
**Fat mass (%)**			
Baseline	35.9 ± 5.8	37.5 ± 4.6	
8-week	34.2 ± 6.0 ^†^	35.7 ± 4.9 ^†^	0.60
Change	1.7 ± 1.4	1.8 ± 1.3	0.66
**Fat-free mass (%)**			
Baseline	63.6 ± 5.4	63.0 ± 5.9	
8-week	65.4 ± 5.7 ^†^	64.82 ± 6.0 ^†^	0.76
Change	−1.7 ± 1.4	−1.8 ± 1.3	0.76

^a^All values are mean ± SD. *BMI* Body mass index, *CCR* Continous calorie restriction diet, *ICR* Intermittent calorie restriction diet

^b^The 8-week values were analyzed using ANCOVA and including baseline values of each variable as a covariate in the model

^c^Change from baseline values were assessed with independent t-tests or non-parametric Manne Whitney U-test

^d^Differences between baseline and post-intervention data were analyzed by the paired *t*-test. Significantly different from Baseline: * *P* < 0.05; ** *P* < 0.01; † *P* < 0.001

^e^Change expressed as the difference between baseline minus week-8 values

### Plasma cardiometabolic markers

Plasma lipid concentrations are displayed in Table 4. From baseline to 8-week, the TG level reduced in both groups (by 15.6 and 6.3% in ICR and CCR groups, respectively) with no significant difference between treatment groups. Total cholesterol and HDL-C concentrations decreased in the CCR group only (*P* < 0.05) but there was no significant difference between the two groups. LDL-C was not affected by either intervention. Non-HDL-C decreased in the CCR group while TG/HDL-C decreased in the ICR group with no difference between treatment groups.

**Table 4:** Plasma levels of risk indicators of cardiovascular disease during the study period ^a^

Outcome	Groups		***P***-value ^**b,c**^
	CCR (***n*** = 44)	ICR (***n*** = 44)	
**TG (mg/dL)**			
Baseline	165.0 (126)	180.5 (115)	
8-week	162 (85) ^d*^	133 (92) ^**^	0.44
Change ^e^	16.0 (113)	23.5 (103)	0.49
**Cholesterol (mg/dL)**			
Baseline	190.1 ± 38.1	178.6 ± 30.3	
8-week	175.7 ± 37.4 ^*^	174.1 ± 30.9	0.57
Change	14.4 ± 37.1	4.56 ± 33.8	0.19
**LDL-C (mg/dL)**			
Baseline	107.7 ± 29.0	98.4 ± 28.5	
8-week	102.9 ± 29.4	103.4 ± 26.1	0.31
Change	4.8 ± 26.2	−5.1 ± 29.1	0.09
**HDL-C (mg/dL)**			
Baseline	42.1 ± 10.6	39.1 ± 7.7	
8-week	39.7 ± 9.3^*^	39.4 ± 6.7	0.28
Change	2.4 ± 7.0	−0.3 ± 7.2	0.08
**Total**: **HDL-C**			
Baseline	4.7 ± 1.2	4.9 ± 1.4	
8-week	4.6 ± 1.1	4.5 ± 1.0	0.73
Change	0.1 ± 1.0	0.3 ± 1.5	0.67
**TG:HDL-C**			
Baseline	5.2 ± 3.0	5.9 ± 4.4	
8-week	4.5 ± 2.4	4.2 ± 2.2 ^*^	0.31
Change	0.7 ± 3.1	1.7 ± 4.3	0.42
**non-HDL -C**			
Baseline	148.0 ± 36.7	139.5 ± 31.4	
8-week	135.9 ± 34.7 ^*^	134.7 ± 30.6	0.69
Change	12.0 ± 35.4	4.8 ± 34.3	0.33
**FBS (mg/dL)**			
Baseline	89.5 (16)	88.0 (26)	
8-week	91.0 (24)	88.0 (15)	0.26
Change	−1.0 (18)	0.0 (21)	0.04
**Insulin (μIU/mL)**			
Baseline	22.0 ± 9.7	18.2 ± 8.1	
8-week	21.3 ± 11.2	16.01 ± 5.5 *	0.17
Change	0.8 ± 6.5	2.1 ± 6.9	0.34
**HOMA-IR**			
Baseline	3.7 (3)	3.5 (3)	
8-week	4.3 (3)	3.5 (2) *	0.03
Change	0.0 (1)	0.3 (1)	0.03
**Adiponectin (μg/mL)**			
Baseline	8.6 ± 3.0	8.1 ± 3.0	
8-week	8.6 ± 2.9	7.9 ± 3.0	0.30
Change	−0.1 ± 2.3	0.3 ± 2.4	0.50
**ALT (IU/L)**			
Baseline	28.2 ± 19.4	20.5 ± 10.9	
8-week	26.7 ± 16.4	19.3 ± 8.2	0.12
Change	1.4 ± 20.6	1.1 ± 11.1	0.82
**AST (IU/L)**			
Baseline	23.3 ± 11.0	19.0 ± 5.5	
8-week	23.0 ± 9.7	19.0 ± 5.1	0.15
Change	0.3 ± 11.8	0.0 ± 5.8	0.96

^a^Values are mean ± SD or median (interquartile range)

^b^The 8-week values were analyzed using ANCOVA and including baseline values of each variable as a covariate in the model

^c^Change from baseline values were assessed with independent t-tests or non-parametric Manne Whitney U-test

^d^Differences between baseline and post-intervention data were analyzed by the paired *t*-test. Significantly different from baseline: * *P* < 0.05; ** *P* < 0.01

^e^Change expressed as the difference between baseline minus week-8 values

Abbreviations: *ALT* alanine aminotransferase, *AST* aspartate aminotransferase, *CCR* Continuous calorie restriction diet, *HDL-C* high-density lipoprotein cholesterol, *HOMA-IR* homeostasis model assessment of insulin resistance [calculated as insulin × glucose/405], *ICR* Intermittent calorie restriction diet, *LDL-C* low-density lipoprotein cholesterol, *TG* triglycerides

There were also no significant differences in fasting plasma glucose between groups at week 8. In the ICR group, the plasma insulin levels decreased significantly from baseline, but the difference between groups was not statistically significant. Participants in the ICR group showed an improvement in HOMA-IR, and the treatment effects were significantly different from those in the CCR group. In addition, the insulin and HOMA-IR were analyzed by considering the baseline weight as well as baseline values of the variables as confounding factors, and no change was observed in the results (*P* = 0.07 and *P* = 0.25 for HOMA-IR and insulin levels, respectively).No changes in the plasma level of adiponectin occurred in either diet group. Plasma aspartate aminotransferase (AST) and alanine aminotransferase (ALT) were not different between groups.

The change values (baseline minus 8-week) of body weight were positively correlated with the corresponding changes in plasma TG (*r =* 0.35; *P* = 0.001) but not with insulin levels. The TG changes were correlated with the changes in insulin levels (*r =* 0.26; *P* = 0.015).

## Discussion

The primary goal of this study was to determine whether subjects with overweight or obesity and mild-to-moderate HTG could benefit from intermittent dieting. The results of this trial demonstrated that three-days a week of the intermittent calorie restriction is comparable to a continuous energy restriction diet for the reduction of plasma TG level and appeared to be more effective than continuous dieting in improving a marker of insulin resistance in this group of participants with HTG.

A slightly greater weight loss was observed in ICR than the CCR diet after 8 weeks of treatment. However, this difference was not significant statistically and it was not also meaningful from the clinical point of view. Consistent with this finding, previous short-term (8–12 weeks) [20, 22, 23] or longer-term [24–26] studies that compared weight loss between the two types of daily and intermittent calorie restriction have not observed a statistically significant difference. It seems that the overall reduction in calorie intake appears to play a major role in weight loss and as long as overall energy restriction remains similar, ICR will result in comparable weight loss to CCR [27].

We observed no significant difference in plasma lipids and lipoproteins between the two groups. Consistent with our findings, a meta-analysis comparing ICR with CCR found no significant between-arms difference in blood lipid values [28]. Beneficial modulations in TG were observed by both diets. Reductions in plasma TG by ICR found in our study are consistent with those reported by several other investigators [25, 29, 30] but they differ from other reports of no change [31]. We also show that changes in plasma TG concentrations were correlated to changes in body weight. Therefore, the size of weight loss may have played a major role in the degree to which the plasma TG was altered. Thus, a slightly greater reduction in plasma TG in the ICR group may be related to their greater weight loss. However, the ICR group had lower carbohydrate intake as well and this may also have contributed to a slightly greater reduction in their plasma TG concentrations. Increases in dietary carbohydrates have been associated with an increase in TG concentration, particularly in overweight and obese subjects [32]. Regarding plasma HDL-C concentration, although no significant difference was found between groups at 8 weeks, the concentration decreased in the CCR group whereas it had no changes in the ICR group. However, it is unlikely this decrease is detrimental because no decrease was observed in the TC/HDL-C. TC/HDL-C has been suggested to be a cumulative marker of the metabolic abnormalities in individuals with high TG–low HDL-C levels [33].

Beneficial modulations in insulin and HOMA-IR (an insulin resistance marker) were observed by the ICR diet. Previous studies have reported either improvement [25, 34] in insulin resistance by ICR compared with CCR or no effect [17, 24, 35]. The effectiveness of the ICR diet may vary according to the study population. The result of the study by Gabel et al. demonstrated that in insulin-resistant participants, ICR produced more improvements in insulin resistance compared with CCR [34]. In contrast, Catenacci et al. noticed no difference in insulin sensitivity index after 8 weeks between ICR and CCR in metabolically healthy individuals with obesity [35]. Likewise, Trepanowski et al. reported no significant differences in fasting insulin or insulin resistance between the ICR and CCR in metabolically healthy obese adults [24]. In another study, individuals who participated in an 8-week ICR diet were divided into tertiles base on the on HOMA-IR [27]. They observed that ICR reduced insulin resistance (estimated by HOMA-IR) only in those that were in the highest tertile of HOMA-IR. In our study, changes in plasma TG concentrations were correlated to changes in insulin. HTG is associated with metabolic glucose disorders [6, 7] and the elevated concentrations of TG levels may lead to peripheral insulin resistance [12]. Subjects with overweight or obesity and HTG of the present study may have more insulin resistance than healthy individuals with overweight or obesity.

Despite a change in insulin resistance, plasma adiponectin levels were not different between ICR or CCR diet groups. This is consistent with the result of previous studies that have compared ICR to CCR [17, 36]. Adiponectin changes during calorie-restriction may have little relation to the amount of weight loss [37].

### Study strengths and limitations

In the present study, the attrition rate was not high, with a dropout rate of only ∼9%. Furthermore, a well-trained dietitian collected the data through face-to-face interviews. However, the study also has a few limitations. First, the duration of the intervention was short and no maintenance phase was included in the study design. Longer trial durations may likely have more clearly shown differences in plasma TG and insulin between groups. Second, there was no run-in period before treatment and participants took their usual diet up to the start of the study. Third, the sample size was not powered to detect differences in cardiometabolic outcomes. In addition, although not statistically significant, at baseline the ICR group had a 6 kg lower body weight than CCR.

## Conclusion

The main finding of our study is that ICR is an effective alternative diet strategy for the reduction of TG level in patients with mild to moderate HTG, and it may be partly superior to CCR for improving insulin resistance in the short-term. There is general agreement that lifestyle therapy, including a weight reduction program, is the cornerstone of treatment for mild-to-moderate HTG [38]. Based on results of the current study, ICR can be recommended for a short term in people with HTG who are reluctant to follow a continuous daily low-calorie diet. However, longer-term studies are needed to confirm these findings before ICR can be recommended more broadly.

## Data Availability

All data analyzed during this study are included in this published article.

## Abbreviations

ALT: Alanine aminotransferase
AST: Aspartate aminotransferase
BMI: Body mass index
CCR: Continuous calorie restriction
HDL-C: High-density lipoprotein cholesterol
HOMA-IR: Homeostasis model assessment of insulin resistance
HTG: Hypertriglyceridemia
LDL-C: Low-density lipoprotein cholesterol
ICR: Intermittent calorie restriction
